# The role of IL-36 and 37 in hepatocellular carcinoma

**DOI:** 10.3389/fimmu.2024.1281121

**Published:** 2024-01-19

**Authors:** Juan Cao, Jun-Hong Liu, Steven G. Wise, Jingchun Fan, Shisan Bao, Gui-Sen Zheng

**Affiliations:** ^1^Basic Medical College, Gansu University of Chinese Medicine, Lanzhou, China; ^2^Department of Public Health, Affiliated Hospital of Gansu University of Chinese Medicine, Lanzhou, China; ^3^Gansu Provincial Integrated Traditional Chinese and Western Medicine Digestive Disease Clinical Research Centre, Lanzhou, China; ^4^School of Medical Sciences, Faculty of Medicine and Health, The University of Sydney, Sydney, NSW, Australia; ^5^School of Public Health, Gansu University of Chinese Medicine, Lanzhou, China

**Keywords:** HCC, IL-36, IL-37, pathogenesis, TAM

## Abstract

Hepatocellular carcinoma (HCC) has garnered considerable attention due to its morbidity and mortality. Although the precise mechanisms underlying HCC tumorigenesis remain to be elucidated, evidence suggests that host immunity plays a pivotal role in its development. IL-36 and IL-37 are important immunoregulatory cytokines classified as pro-inflammatory and anti-inflammatory respectively. In the context of HCC, the downregulation of intrahepatic IL-36 is inversely correlated with cirrhosis, but positively correlated with 5-year survival rates, suggesting that IL-36 offers protection during HCC development. However, IL-36 may lose its hepatoprotective effects as the disease progresses to HCC in the context of dysregulated immunity in cirrhotic patients. Substantially increased circulating IL-36 in HCC patients is likely a systemic response to HCC stimulation, but is insufficient to suppress progression towards HCC. Intrahepatic IL-37 is suppressed in HCC patients, consistent with the inverse correlation between intrahepatic IL-37 and the level of AFP in HCC patients, suggesting IL-37 exerts hepatoprotection. There is no significant difference in IL-37 among differentiations of HCC or with respect to clinical BCLC stages or cirrhosis status in HCC patients. However, IL-37 protection is demonstrated in an IL-37 transfected HCC animal model, showing significantly reduced tumour size. IL-36/37 may inhibit HCC by enhancing M1 tumour-associated macrophages while not affecting M2 macrophages. The interplay between IL-36 (pro-inflammatory) and IL-37 (anti-inflammatory) is emerging as a crucial factor in host protection against the development of HCC. Further research is needed to investigate the complex mechanisms involved and the therapeutic potential of targeting these cytokines in HCC management.

## Hepatocellular carcinoma

Hepatocellular carcinoma (HCC) represents the most prevalent form of primary liver cancer and is associated with high mortality and morbidity (1). A significant proportion of HCC patients receive palliative care due to late-stage diagnosis characterized by distant and multiple metastases. Disturbingly, projections indicate that global HCC-related deaths will reach 8 million by 2050 (2). The pathogenesis of HCC is multifaceted, involving hepatic damage occurring in genetically susceptible individuals because of intricate interactions between hepatic damage and host immunity within the microenvironment (2). The aetiology of HCC is influenced by regional and racial factors, with persistent hepatic damage from various sources such as viral, alcoholic, non-alcoholic steatohepatitis, or drug-induced liver injury serving as precursors for the progression towards cirrhosis and subsequent development of HCC (3). Host immunity plays a critical role in the pathogenesis of malignancies, as evidenced by the early successes of immune checkpoint blockade therapy (4) in reactivating compromised host immune responses. However, the application of immune checkpoint blockade in HCC patients has been associated with serious adverse reactions (5, 6), including severe hepatic damage (7). Consequently, gaining a better understanding of the underlying mechanisms involved in HCC development is imperative for designing personalized precision medicine approaches that can mitigate these adverse effects and improve patient outcomes.

## The physiological and pathological role of IL-36 and IL-37

### IL-36

IL-36, a member of the IL-1 superfamily, shares sequence homology (8) and is widely expressed in multiple cell types, including keratinocytes, endothelial cells, and various immune cells (9). Epithelial cells from the lung, intestine, and skin, as well as monocytes and myeloid dendritic cells, are known to produce IL-36 (10). IL-36 exerts its effects on cells and tissue surfaces through the IL-36 receptor, utilizing classical NF-κB, MAPKs, JNK, and ERK1/2 kinase cascades (10). Functionally, IL-36 is considered a pro-inflammatory cytokine, as its agonists promote the production of pro-inflammatory cytokines and chemokines, such as IL-17C, IL-8, G-CSF, CXCL-1, and CCL-20 (11), thereby enhancing inflammatory responses (10). Elevated levels of IL-36 have been observed in various autoimmune diseases, including psoriatic arthritis, systemic lupus erythematosus (12), Sjogren’s syndrome (10), and inflammatory bowel disease (12, 13), indicating its significant role in host immunity. Moreover, studies have demonstrated distinct roles for different IL-36 isoforms (IL-36α, β, γ) in the development of colorectal cancer (14), while IL-36γ has been shown to promote the growth of stomach cancer (14), indicating the complex involvement of IL-36 in carcinogenesis. Notably, the close correlation between IL-36 and the prognosis of colorectal cancer (10), but an inverse correlation with gastric cancer (14), suggests that IL-36 may hold promise as a therapeutic target for cancer management. Promising outcomes have been observed in animal models of induced fibrosarcoma following IL-36 gene therapy (12). The biological role of a closely related yet anti-inflammatory cytokine, IL-37, will be described below.

### IL-37

IL-37, another member of the IL-1 superfamily, was initially designated as IL-1 family member 7 (IL-1F7) due to being the seventh discovered member of the IL-1 family (15). It shares the structural characteristics typical of the IL-1 family (16). The IL-37 gene spans 3.617 kb, consisting of six exons that encode a protein weighing between 17 and 26 kDa (17). Constitutive production of IL-37 has been observed in various cell types, including leukocytes (such as NK cells, activated B cells, and monocytes), epithelial cells (including keratinocytes and epithelial cells), and other tissues like the lung, intestine, uterus, lymphoid tissues (lymph nodes and thymus) (18, 19). The unique feature of IL-37 is its ability to inhibit both innate (16) and adaptive immunity (20), thereby suppressing the host response (21) for protective purposes, leading to its classification as an anti-inflammatory cytokine. This classification is supported by findings demonstrating that IL-37 mitigates the progression of atherosclerosis in an *in vivo* IL-37 transgenic animal model (22). The present study provides a review of the roles of IL-36 and IL-37 in HCC.

### IL-36 in HCC

In non-cancerous liver tissues, there is a constitutive level of intrahepatic IL-36; however, the expression of intrahepatic IL-36 is significantly suppressed in HCC tissues (23). Notably, HCC patients with higher intrahepatic IL-36 expression exhibit better survival outcomes, along with lower recurrence rates, compared to HCC patients with lower intrahepatic IL-36 expression (23). These findings suggest that IL-36 plays a protective role during HCC development, possibly by enhancing inflammation within the focal lesion (24). However, overall host immunity is not sufficiently strong among susceptible individuals to completely eliminate HCC cells, leading to the progression of the disease.

Interestingly, the level of circulating IL-36 increases gradually from low to high in patient cohorts comprising HCC, chronic hepatitis B, and healthy controls (23). Flow cytometric analysis reveals that the frequency of IL-36^+/^CD4^+^ cells and IL-36^+^/CD8^+^ cells in peripheral blood mononuclear cells is doubled in HCC patients compared to healthy controls or patients with chronic hepatitis B (23). These observations suggest that the elevated circulating IL-36 in HCC patients mainly originates from circulating CD4^+^ T helper and CD8^+^ cytotoxic T cells, rather than from intrahepatic lymphocytes and/or hepatocytes. This phenomenon may reflect the host’s attempt to provide protection against HCC malignancy through IL-36-mediated pro-inflammatory responses, which may be a result of or a response to persistent stimulation from HCC cancer cells. However, the overall effectiveness of direct cytotoxic T cell killing and/or indirect antibody-mediated killing of mutated malignant cells is compromised in these susceptible patients, eventually leading to disease progression (24).

Cirrhosis represents a significant risk factor for the development of HCC (25), and therefore, the association between IL-36 and cirrhosis was further investigated. It was observed that there is an inverse correlation between intrahepatic IL-36 levels and cirrhosis in HCC patients (23). Considering that host immunity is dysregulated and compromised in cirrhotic patients (26), HCC patients with cirrhosis may attempt to eliminate malignant cells by secreting IL-36 to enhance cell-mediated direct killing and/or antibody-dependent cytotoxicity during the transition from cirrhosis to HCC. However, their compromised host immunity fails to initiate an effective defence against malignancy. Furthermore, the correlation between IL-36 and the prognosis of HCC (23) provides additional support for the protective role of IL-36 in HCC. The observed inverse correlation between IL-36 and metastasis (23) may be attributed to the insufficient ability of the host to inhibit distant migration. Additionally, it remains unclear whether there is impairment of the downstream IL-36 pathway and/or IL-36 receptor on the targeted HCC cells, which will require further investigation for clarification in future studies.

There is a lower portion of IL-36 positive HCC patients eligible for radical surgical resection of the tumours than IL-36 negative HCC cohort (23), reflecting the correlation between the advanced stages of disease and IL-36 production in the liver, or loss of IL-36 local protection. This is supported by the finding that HCC patients undergoing transarterial chemoembolisation (TACE) presented a lower portion of IL-36 positive, compared to those without TACE (23). HCC patients selected for TACE usually present with multiple and/or large size tumours who may not be eligible for radical resection, which is in line with observed substantially reduced intra-hepatic IL-36 from HCC patients (27).

More recently, a report has brought attention to an inverse correlation between the levels of circulating and intra-hepatic IL-36α and the differentiation of hepatocellular carcinoma (HCC) (28). This observation suggests a potential protective role for IL-36α during HCC development. Bioinformatics analysis has identified miR-27b-3p as closely associated with downstream IL-36α. Given that miR-27b-3p reverses multi-drug resistance in breast cancer by targeting CBLB/GRB2 (29) *in vitro* and *in vivo*, the expression of miR-27b-3p in HCC tissues was investigated, revealing an upregulation that displayed an inverse relationship with IL-36α in HCC, potentially mediated through CXCL1 in HCC cells (28). Further confirmatory studies indicated that IL-36α exerts inhibitory effects on HCC proliferation, viability, and migration *in vitro*. These findings align with a concurrent reduction in the expression of pro-inflammatory cytokines (IL-1β and IL-18), suggesting a potential role for IL-36α in inhibiting pyroptosis (28).

The primary source of intrahepatic IL-36 in liver injury has been demonstrated to be infiltrating neutrophils following Con A challenge *in vivo* (30). The hepatoprotective function of the IL-36 pathway has been established through studies showing that the depletion of IL-36R leads to exacerbated liver injury (30). This injury is characterized by increased mortality, elevated serum levels of ALT/AST, and significant pathological changes (30). Additionally, there is a substantial increase in intrahepatic activation of CD4+ and CD8+ T lymphocytes, accompanied by an escalation in the production of inflammatory cytokines. Moreover, IL-36R-/- mice showed reduced numbers of T regulatory cells, indicating the crucial involvement of IL-36 in modulating T cell function and maintaining homeostasis during liver inflammation (30).

However, there is room for improvement in the studies above. First, IL-36 can be divided into three subunits, i.e. IL-36α, β, γ (10), which have quite distinctive roles during the development of cancer. For example, it has been illustrated that IL-36α and γ, but not β, augments the development of colorectal cancer (28), showing an inverse correlation between IL-36α and γ expression and five-year survival of colorectal cancer patients. In addition, the quality of the immunostaining for IL-36 in the HCC study is not high enough (23), which likely compromises the quantification of data and subsequent statistics to some degree. Therefore, better immunohistochemical staining with the three subsets and consequent computerised automatic quantification is desirable in the future (29, 30). A large cohort with different genetic backgrounds and a multicentre study would also be useful in confirming the differential roles of IL-36α, β, γ during the development of HCC and for exploring the potential role of these three subsets in the design of personalised precision medicine approaches to boost outcomes for HCC patients, particularly for patients in the advanced stage who are currently only provided palliative care (31).

### IL-37 in HCC

There is substantially reduced intra-hepatic IL-37 from the tumour tissues, compared to adjacent non tumour tissue of HCC (31), which is consistent with the inverse correlation between IL-37 and alfa foetal protein (AFP) levels from HCC patients (31), both supporting a protective role for IL-37 during the development of HCC. AFP is a reasonably reliable, versatile, and low economic cost biomarker for detection of HCC used by clinicians to make management decisions over the last decades (32). The hepato-protective role of IL-37 is illustrated in animal models, showing that exogenous IL-37 ameliorates hepatocyte damage in an ischemia/reperfusion model, accompanied by reduced pro-inflammatory cytokines, e.g. TNF (33) via upregulating the Bcl-2 pathway (33). It should be emphasised that activation of the Bcl-2 signalling pathway is critically important in carcinogenesis, including for HCC (34), which is consistent with poor prognosis. Furthermore, IL-37 supresses HCC via activating and recruiting dendritic cells during the development of HCC (35).

Initial evaluation of tissue from HCC patients did not reveal a significant difference in intra-hepatic IL-37 levels between older (>55 years) and younger (<55 years) cohorts or between male and female patients (31). However, an important limitation of this study is that the cut-off age of 55 may overlook the potential protective effects of sex hormones in HCC development. Furthermore, no significant difference in intra-hepatic IL-37 levels was observed between tumours smaller than 2 cm and those larger than 2 cm. This lack of significant difference could be attributed to the relatively small sample size (only 65 patients in total) and the fact that the study was conducted at a single centre (31). Surprisingly, no significant differences in IL-37 levels were found among poorly, moderately, and well-differentiated HCC, as well as with respect to clinical BCLC stages or cirrhosis status (31). These findings may also be influenced by the relatively small sample size, limiting the ability to detect significant associations.

Clinical observations align with experiments conducted on HCC cell lines *in vitro*, revealing that IL-37-transfected HepG2 HCC cells significantly suppress the expression of NFκB (31), which plays a crucial role in hepatic injury, fibrosis, and HCC (36) by regulating inflammation and cell death, as emphasized by studies demonstrating that its depletion leads to spontaneous liver injury, fibrosis, and HCC (37).

To explore the underlying anti-HCC mechanism, human and murine HCC cell lines were transfected with IL-37 for overexpression (38). This revealed that tumour-derived IL-37 promotes HUVEC apoptosis, inhibits migration, and impedes tubule formation *in vitro*. These findings align with reduced angiogenesis in the tumour when IL-37-transfected HCC cells are inoculated into the animal model *in vivo* (37). Furthermore, reduced angiogenesis is corroborated by the inhibition of MMP2 and MMP9, potent inducers of VEGF (38).

IL-37 enhances the expression of antiangiogenic factors while concurrently reducing the expression of proangiogenic factors by tumour cells, thereby inhibiting tumoral angiogenesis within the tumour microenvironment (38). The balance between pro-angiogenic and anti-angiogenic factors is tightly controlled to maintain normal physiological homeostasis. Disruption in this balance, with aberrations in angiogenic stimulators and inhibitors, can potentially lead to infectious disorders, inflammation, and even carcinogenesis (38). Thus, targeting the angiogenic switch, either directly or indirectly, emerges as a promising therapeutic strategy for cancer.

Interestingly, when examining the expression of intrahepatic IL-37 in adjacent non-tumour tissues, a significant difference in IL-37 levels was observed between male and female HCC patients. This difference may be attributed to the relatively stronger host immunity in female patients, allowing for a more effective defence against mutated tissues/cells. However, despite these efforts, the overall defence is ultimately unsuccessful, as reflected by the ineffectiveness of host immunity in the tumour microenvironment leading to HCC development. This finding aligns with previous studies demonstrating substantially suppressed IL-37 expression in HCC tissue (39). In addition, higher intrahepatic IL-37 expression is strongly correlated with improved overall survival and disease-free survival, providing further support for the protective role of IL-37 in HCC. Additionally, a positive correlation exists between intrahepatic IL-37 levels and infiltrating CD57^+^ NK cells within the HCC tissues, consistent with the ability of exogenous IL-37 to recruit CD57^+^ NK cells *in vitro* (39). CD57^+^ NK cells are mature, terminally differentiated cells with high cytotoxicity, and their recruitment helps directly eliminate malignant cells. This observation is further substantiated by the significant inhibition of HCC growth observed in IL-37 transgenic HCC models, which is accompanied by reduced CD57^+^ NK cell presence *in vivo* (39).

Furthermore, the findings from HCC align with observations in colorectal cancer, where both mRNA and protein levels of IL-37 are substantially reduced in colorectal cancer tissues compared to non-cancer tissues (40). These similarities suggest a shared host immunity response between the liver and colon, as both organs are covered with mucosal epithelial cells and protected by mucosal-associated lymphoid tissues.

## Tumour associated macrophages

It is a subject of ongoing debate whether a pro- or anti-inflammatory response is beneficial during the development of malignancy. Local pro- or anti-inflammatory responses can influence the polarization of M0 macrophages, leading to their differentiation into either classical M1 macrophages (pro-inflammatory) or M2 alternative macrophages (anti-inflammatory). The function of these macrophage subsets can either inhibit or promote cancer development. In the case of HCC, the protective role of IL-37 in the liver is evident. IL-37 has been shown to enhance the polarization of M1 macrophages, which in turn promotes anti-cancer activity (41). This supports the notion that IL-37 plays a hepato-protective role during HCC development by influencing the immune response and promoting an anti-cancer environment.

Extensive infiltration of various types of leukocytes has been well-documented in HCC tissues, including T and B lymphocytes, neutrophils, NK cells, and macrophages (42). In this mini-review, we specifically focus on tumour-associated macrophages (TAMs) within HCC tissues. However, there is an ongoing debate regarding the beneficial or detrimental effects of TAMs in HCC (43), with the outcome likely dependent on their terminal differentiation. TAMs can be classified into two main subsets based on their terminal differentiation: classically activated M1 macrophages or alternatively activated M2 macrophages (44). The functional role of TAMs in HCC remains controversial, as their effects on tumour growth and progression can be diverse.

M1 TAMs possess the ability to directly kill tumour cells through the release of reactive oxygen species (ROS) and nitric oxide (NO). They can also contribute to antibody-dependent cell-mediated cytotoxicity (ADCC), which aids in tumour cell destruction. Conversely, M2 TAMs promote tumour development by potentially inhibiting T cell-mediated anti-tumour immune responses and promoting tumour angiogenesis, thereby facilitating tumour progression (44). Recent studies have shown significantly lower levels of IL-37 in peripheral blood mononuclear cells from HCC patients compared to non-cancer cohorts, indicating a reduction in M1 TAMs (45).

TAMs from HCC patients, predominantly consist of M2 TAMs (45). In *in vitro* experiments, TAMs from HCC patients, predominantly consisting of M2 TAMs, demonstrated a polarization into M1 TAMs upon IL-37 transfection (45). This shift led to an increased production of IL-12, TNF, and IL-1β, supporting the anti-tumour role of IL-37. Conversely, the *in vitro* reduction of IL-37 resulted in outcomes of an opposite nature (45). The modulation of IL-37 expression in TAMs conditioned by HCC exerted regulatory effects on the proliferation, migration, and invasion of HepG2 and Huh-7 cells.

Furthermore, in an HCC transplantation model *in vivo*, the inoculation of IL-37-transfected TAMs into recipients resulted in a substantial reduction in tumour growth compared to mice without IL-37 transfection (43), providing additional confirmation of the anti-tumour function of IL-37. Moreover, in the same HCC transplantation model *in vivo*, inoculating IL-37-transfected TAMs into the recipients resulted in significantly reduced tumour growth compared to mice without IL-37 transfection (45), providing further confirmation of the anti-tumour function of IL-37.

However, it is important to note that the recipients in this study were nude mice, which lack host immunity. Therefore, future investigations should consider using humanized animals or immunocompetent hosts with human HCC cells or manipulated IL-37 (knockout or transgenic) animals to verify the anti-tumour function *in vivo*. This will help explore the potential of IL-37 in HCC management, potentially through the modulation of M1 and M2 macrophages.

The utilization of anti-PD-1/PD-L1 treatments in the context of Hepatocellular Carcinoma (HCC) has received extensive coverage (46). These treatments are often employed in both monotherapy and in combination with anti-angiogenic agents. Despite an exhaustive search, we could not uncover any existing literature that explores the direct connections among IL-36, IL-37, HCC, and the PD-1/PD-L1 axis. Nevertheless, considering the acknowledged immunological functions of IL-36 and IL-37 in the context of HCC, as mentioned above, it is conceivable that the targeting of T cells through anti-PD-1/PD-L1 therapies could potentially influence the production of IL-36 and/or IL-37, thereby yielding downstream effects. The precise nature of these effects remains a subject for future investigation and will require further elucidation.

The source of IL-36 can be traced to intrahepatic CD4+/CD8+ T cells in HCC patients, as well as in CD4+/CD8+ T cells from peripheral blood mononuclear cells (PBMC). Notably, there is a substantial increase in IL-36 production observed in HCC patients when compared to non-HCC individuals, as reported by Hu (23), which may be linked to an unsuccessful anti-viral response. Furthermore, IL-37 is primarily expressed in circulating monocytes, tissue macrophages, dendritic cells (DCs), tonsil B cells, and plasma cells, as noted in the study by (47). As previously discussed, the roles of both IL-36 and IL-37 in HCC have been well-documented.

These findings prompt speculation about potential clinical implications for the development of novel drugs. One intriguing avenue may involve enhancing IL-36 and/or IL-37 production by bolstering T cell activities in combination with anti-PD-1 treatment, opening up promising avenues for future research and therapeutic strategies.

In conclusion, both IL-36 and IL-37 play important roles in the inflammatory response, with IL-36 being pro-inflammatory and IL-37 being anti-inflammatory. Interestingly, both cytokines appear to provide hepato-protection during the development of HCC, regardless of its underlying cause. This knowledge holds promise for the development of novel therapeutic interventions that can effectively manage HCC while minimizing adverse effects. By targeting the specific pathways and mechanisms involving IL-36 and IL-37, it may be possible to achieve improved outcomes and better patient prognosis in HCC treatment.

## Author contributions

JC: Conceptualization, Writing – original draft. J-HL: Conceptualization, Writing – review & editing. SW: Writing – review & editing. JF: Writing – review & editing, Conceptualization. SB: Conceptualization, Writing – review & editing. G-SZ: Supervision, Writing – review & editing.
